# Loss of tenomodulin expression is a risk factor for age‐related intervertebral disc degeneration

**DOI:** 10.1111/acel.13091

**Published:** 2020-02-21

**Authors:** Dasheng Lin, Paolo Alberton, Manuel Delgado Caceres, Carina Prein, Hauke Clausen‐Schaumann, Jian Dong, Attila Aszodi, Chisa Shukunami, James C Iatridis, Denitsa Docheva

**Affiliations:** ^1^ Experimental Surgery and Regenerative Medicine Clinic for General, Trauma and Reconstructive Surgery Ludwig‐Maximilians‐University (LMU) Munich Germany; ^2^ Orthopaedic Center of People’s Liberation Army The Affiliated Southeast Hospital of Xiamen University Zhangzhou China; ^3^ Experimental Trauma Surgery Department of Trauma Surgery University Regensburg Medical Centre Regensburg Germany; ^4^ Center for Applied Tissue Engineering and Regenerative Medicine (CANTER) Munich University of Applied Sciences and Center for Nanoscience (CeNS) Munich Germany; ^5^ Department of Orthopaedic Surgery Zhongshan Hospital Fudan University Shanghai China; ^6^ Department of Molecular Biology and Biochemistry Graduate School of Biomedical & Health Sciences Hiroshima University Hiroshima Japan; ^7^ Leni and Peter W. May Department of Orthopaedics Icahn School of Medicine at Mount Sinai New York NY USA

**Keywords:** angiogenesis, annulus fibrous, intervertebral disc degeneration, knockout mice, nucleus pulposus, tenomodulin

## Abstract

The intervertebral disc (IVD) degeneration is thought to be closely related to ingrowth of new blood vessels. However, the impact of anti‐angiogenic factors in the maintenance of IVD avascularity remains unknown. Tenomodulin (*Tnmd*) is a tendon/ligament‐specific marker and anti‐angiogenic factor with abundant expression in the IVD. It is still unclear whether Tnmd contributes to the maintenance of IVD homeostasis, acting to inhibit vascular ingrowth into this normally avascular tissue. Herein, we investigated whether IVD degeneration could be induced spontaneously by the absence of *Tnmd*. Our results showed that Tnmd was expressed in an age‐dependent manner primarily in the outer annulus fibrous (OAF) and it was downregulated at 6 months of age corresponding to the early IVD degeneration stage in mice. *Tnmd* knockout (*Tnmd* ^−^ *^/^* ^−^) mice exhibited more rapid progression of age‐related IVD degeneration. These signs include smaller collagen fibril diameter, markedly lower compressive stiffness, reduced multiple IVD‐ and tendon/ligament‐related gene expression, induced angiogenesis, and macrophage infiltration in OAF, as well as more hypertrophic‐like chondrocytes in the nucleus pulposus. In addition, *Tnmd* and chondromodulin I (*Chm1*, the only homologous gene to *Tnmd*) double knockout (*Tnmd* ^−^ *^/^* ^−^ *Chm1* ^−^ *^/^* ^−^) mice displayed not only accelerated IVD degeneration, but also ectopic bone formation of IVD. Lastly, the absence of *Tnmd* in OAF‐derived cells promoted p65 and matrix metalloproteinases upregulation, and increased migratory capacity of human umbilical vein endothelial cells. In sum, our data provide clear evidences that Tnmd acts as an angiogenic inhibitor in the IVD homeostasis and protects against age‐related IVD degeneration. Targeting Tnmd may represent a novel therapeutic strategy for attenuating age‐related IVD degeneration.

## Graphical abstract

Cartoon highlighting the hallmarks of Tnmd^−/−^ intervertebral disc (IVD) phenotype. Tnmd^−/−^ mice exhibited more rapid progression of age‐related IVD degeneration. These signs include lower disc height index, smaller collagen fibrils with lower compressive stiffness in the outer annulus fibrosus, reduced multiple IVD‐ and tendon/ligament‐related gene expression, induced angiogenesis and macrophage infiltration in outer annulus fibrous, as well as more hypertrophic‐like chondrocytes in the nucleus pulposus.

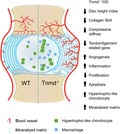

## 1 INTRODUCTION

Intervertebral disc (IVD) degeneration is a common condition and is thought to be an initiating factor for back pain (Nguyen, Poiraudeau, & Rannou, 2015). The pathogenesis of IVD degeneration is a complex, multifactorial process with large contribution from both genetic and environmental components (Annunen et al., 1999; Pye, Reid, Adams, Silman, & O'Neill, 2007; Song et al., 2013; Stokes & Iatridis, 2004; Williams et al., 2013; Williams et al., 2011). The IVD is the largest avascular tissue in the body and has poor self‐healing potential (Huang, Urban, & Luk, 2014). Under pathological conditions, the IVDs express pro‐angiogenic factors leading to neovascularization (Cornejo, Cho, Giannarelli, Iatridis, & Purmessur, 2015; de Vries, van Doeselaar, Meij, Tryfonidou, & Ito, 2018; Freemont et al., 1997; Purmessur, Freemont, & Hoyland, 2008). However, the impact of anti‐angiogenic factors in the maintenance of IVD avascularity remains unknown.

Tenomodulin (Tnmd), a tendon/ligament‐specific marker and anti‐angiogenic molecule, is a member of a novel class protein family of type II transmembrane glycoproteins containing only one other homologous protein, namely chondromodulin I (Chm1) that is abundant in cartilage tissue (Brandau, Meindl, Fässler, & Aszódi, 2001; Dex, Lin, Shukunami, & Docheva, 2016; Docheva, Hunziker, Fässler, & Brandau, 2005; Kimura et al., 2008; Shukunami, Oshima, & Hiraki, 2001). The cleavage of the highly conserved C‐terminal cysteine‐rich domain of Tnmd and subsequent secretion are required for the anti‐angiogenic activities of the protein (Oshima et al., 2004). *Tnmd* transcript is predominantly expressed in hypovascular tissues, such as tendons, ligaments, as well as eyes (Brandau et al., 2001; Shukunami et al., 2001). Interestingly, Minogue, Richardson, Zeef, Freemont, and Hoyland (2010) have demonstrated that *Tnmd* mRNA has abundant expression in the annulus fibrous (AF) and the nucleus pulposus (NP). Concomitantly, Nakamichi et al. (2016) showed that *Mohawk* (*Mkx*), an upstream gene of *Tnmd* (Ito et al., 2010), promotes the maintenance and regeneration of the outer annulus fibrous (OAF) of IVD suggesting that *Tnmd* may be involved in IVD homeostasis. To date, however, this hypothesis has not been investigated in detail.

In our previous studies, we compared *Tnmd* knockout (*Tnmd* ^−^ *^/^* ^−^) mice with their wild‐type (WT) littermates and showed that the absence of *Tnmd* causes reduced tendon cell proliferation and density in vivo (Docheva et al., 2005), coupled with significantly lower self‐renewal and augmented senescence of tendon‐derived stem/progenitor cells (TSPCs) in vitro (Alberton et al., 2015). Furthermore, we observed the pathological thickening and stiffening of collagen I (Col I) fibers in *Tnmd* ^−^ *^/^* ^−^ Achilles tendons resulting in running distance and time failure in *Tnmd* ^−^ *^/^* ^−^ mice challenged by running tests (Dex et al., 2017). Interestingly, the local absence of *Tnmd* in the cardiac chordae tendineae cordis (CTC) promoted angiogenesis and matrix metalloproteinases (MMPs) activation (Kimura et al., 2008), a phenomenon also observed when *Tnmd* ^−^ *^/^* ^−^ mice were subjected to surgically induced Achilles tendon rupture. In this model, we detected that genetic ablation of *Tnmd* leads to blood vessel accumulation accompanied by abnormal extracellular matrix (ECM) composition and macrophage profile during the early repair phase of injured tendons (Lin et al., 2017).

Cumulatively, the aforementioned data reveal that *Tnmd* plays an important regulatory role in the avascular tendogenic/ligamentogenic tissue homeostasis. Therefore, we hypothesized that Tnmd in the IVD may act to inhibit vascular ingrowth into this normally avascular tissue and maintain homeostasis. Here, we investigated the exact functional role of Tnmd in IVD in vivo and in vitro by phenotypization of *Tnmd*‐deficient IVD tissues and IVD‐derived cells. Lastly, to rule out possible compensatory effects between the two homologs, we investigated the IVDs of *Tnmd* and *Chm1* double knockout (*Tnmd* ^−^ *^/^* ^−^ *Chm1* ^−^ *^/^* ^−^) mice.

## 2 RESULTS

### 2.1 Tnmd is expressed in the IVD OAF and NP

**Figure 1:**
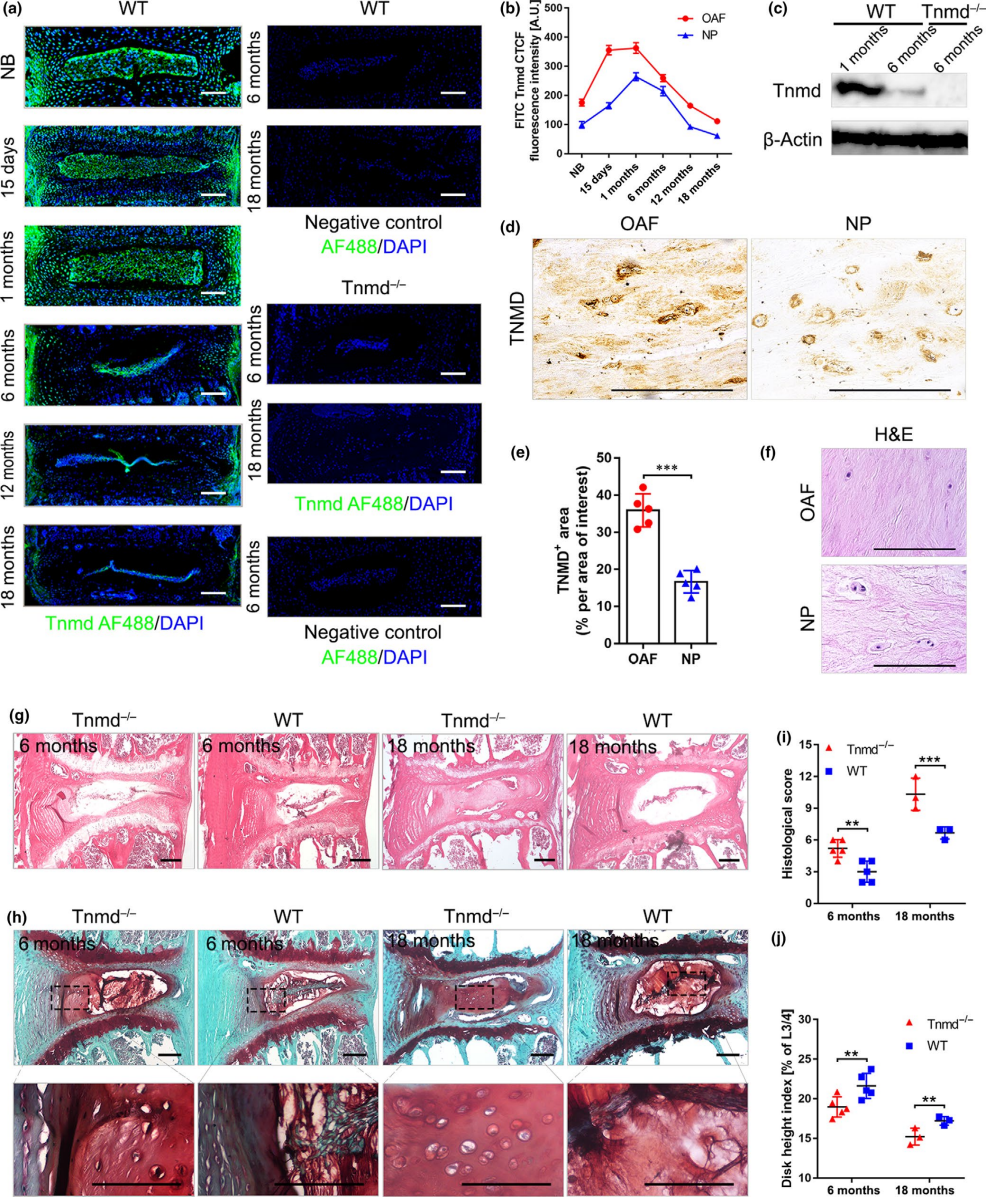
Tnmd is mainly expressed in OAF, and its loss leads to age‐related IVD degeneration. (a and b) Immunofluorescence of Tnmd protein expression in developing adult and aged IVDs of WT mice shows that Tnmd is mainly expressed in OAF and to a lower extent in the NP, and as expected, Tnmd was not detected in *Tnmd^−/−^* IVDs. Fluorescence intensity analysis revealed an expression peak at 1 month and expression downregulation from 6 months onwards (*n* = 3–5 animals). (c) Western blot confirmed that Tnmd protein expression is higher at 1 month than at 6 months (*n* = 3 animals). (d and e) IHC of the human IVDs confirmed that TNMD protein is mainly found in the OAF and to a lesser extended in the NP (two‐tailed nonparametric Mann–Whitney test; *n* = 5 samples). (f) Representative H&E stainings of human IVD (*n* = 5 samples). (g) H&E staining demonstrates greater degenerative changes in *Tnmd^−/−^* IVDs when compared to WT at 6 and 18 months. (h) Safranin O staining reveals small roundish chondrocyte‐like cells in IAF and NP of *Tnmd^−/−^* IVDs. (i and j) Histological grading and disc height index calculation show in *Tnmd^−/−^* mice significantly widespread degeneration compared to WT at both examined stages (two‐tailed nonparametric Mann–Whitney test; 6‐month‐old mice, *n* = 5 animals; 18‐month‐old mice, *n* = 3 animals). ^**^ *p* < .01; ^***^ *p* < .001. d, day; H&E, hematoxylin–eosin; IAF, inner annulus fibrous; IHC, immunohistochemistry; IVD, intervertebral disc; mo, month; NB, newborn; NP, nucleus pulposus; OAF, outer annulus fibrous; WT, wild‐type. Scale bar, 200 μm

Tnmd expression was observed by us, along with other researchers, in tendons, ligaments, eyes, and CTC (Brandau et al., 2001; Kimura et al., 2008; Shukunami et al., 2001). In the vertebral column, previous studies have localized *Tnmd* gene expression to areas of the IVD (Minogue et al., 2010; Nakamichi et al., 2016), as well as Tnmd immunostainings carried out in neonate mice detected robust protein expression in the OAF (Yoshimoto et al., 2017). To further determine the precise distribution of Tnmd in the postnatal and adult IVD, we first performed immunolocalization studies on IVD tissues from WT mice at distinct stages of skeletal development ranging from newborn to 18 months of age. We observed that Tnmd is predominantly produced and deposited in the ECM of the OAF, as well as to a lesser extent in the NP regions. Scarce positive signals in the inner annulus fibrous (IAF) and the cartilaginous endplate (EP) were also detected; however, those were primarily cellular and not in the ECM (Figure 1a). Notably, Tnmd signals in the OAF and the NP gradually peaked at 1 month of age, while it dropped at 6 months of age corresponding to the early IVD degeneration stage in mice, and then further decreased at 12 and 18 months (Figure 1b). As expected, Tnmd was not detected in *Tnmd* ^−^ *^/^* ^−^ IVDs (Figure 1a). Western blotting of IVD tissue protein extracts confirmed that the protein levels of Tnmd are higher at 1 month, when compared to 6 months (Figure 1c) as well as that in the IVD; similar to Achilles tendon and CTC, Tnmd is fully processed to its 16 kDa C‐terminal portion. We also assessed the expression of TNMD in human lumbar discs (Table S1). Consistent with the observation in mice, TNMD protein was predominantly found in the ECM of the OAF and to a lesser extent in the NP regions (Figure 1d–f).

### 2.2 The absence of Tnmd leads to age‐related IVD degeneration

To analyze the potential involvement of Tnmd during naturally occurring IVD degeneration in mouse, 6‐ and 18‐month‐old lumbar IVDs were first examined by hematoxylin–eosin (H&E) and safranin O staining for pathological changes. Importantly, *Tnmd* ^−^ *^/^* ^−^ mice showed higher levels of degenerative changes compared to WT mice. Lamellae appeared thinner, looser, and fibrillated in *Tnmd* ^−^ *^/^* ^−^ AF (Figure 1g). In the IAF and NP areas of *Tnmd* ^−^ *^/^* ^−^ IVDs, small round cells, morphologically resembling chondrocytes, were visible. Cumulatively, such abnormalities are often described as degenerative changes (Nakamichi et al., 2016), and they were even more evident in the *Tnmd* ^−^ *^/^* ^−^ discs at 18 months (Figure 1h). By implementing the histological scoring system for mouse IVD (Tam et al., 2018), we detected higher degenerative scores at 6 and 18 months in *Tnmd* ^−^ *^/^* ^−^ mice, reflective of a significantly widespread IVD degeneration compared to WT (Figure 1i). In addition, calculation of disc height index (Masuda et al., 2005) showed lower values for *Tnmd* ^−^ *^/^* ^−^ IVDs at both examined stages (Figure 1j). Thus, our results demonstrate that Tnmd is associated with IVD homeostasis and its loss leads to profound tissue degeneration that advances during the aging process.

### 2.3 Tnmd deficiency results in abnormal diameters and biomechanical properties of IVD collagen fibrils, accompanied by reduced expression of multiple IVD‐ and tendon/ligament‐related genes in the OAF

**Figure 2:**
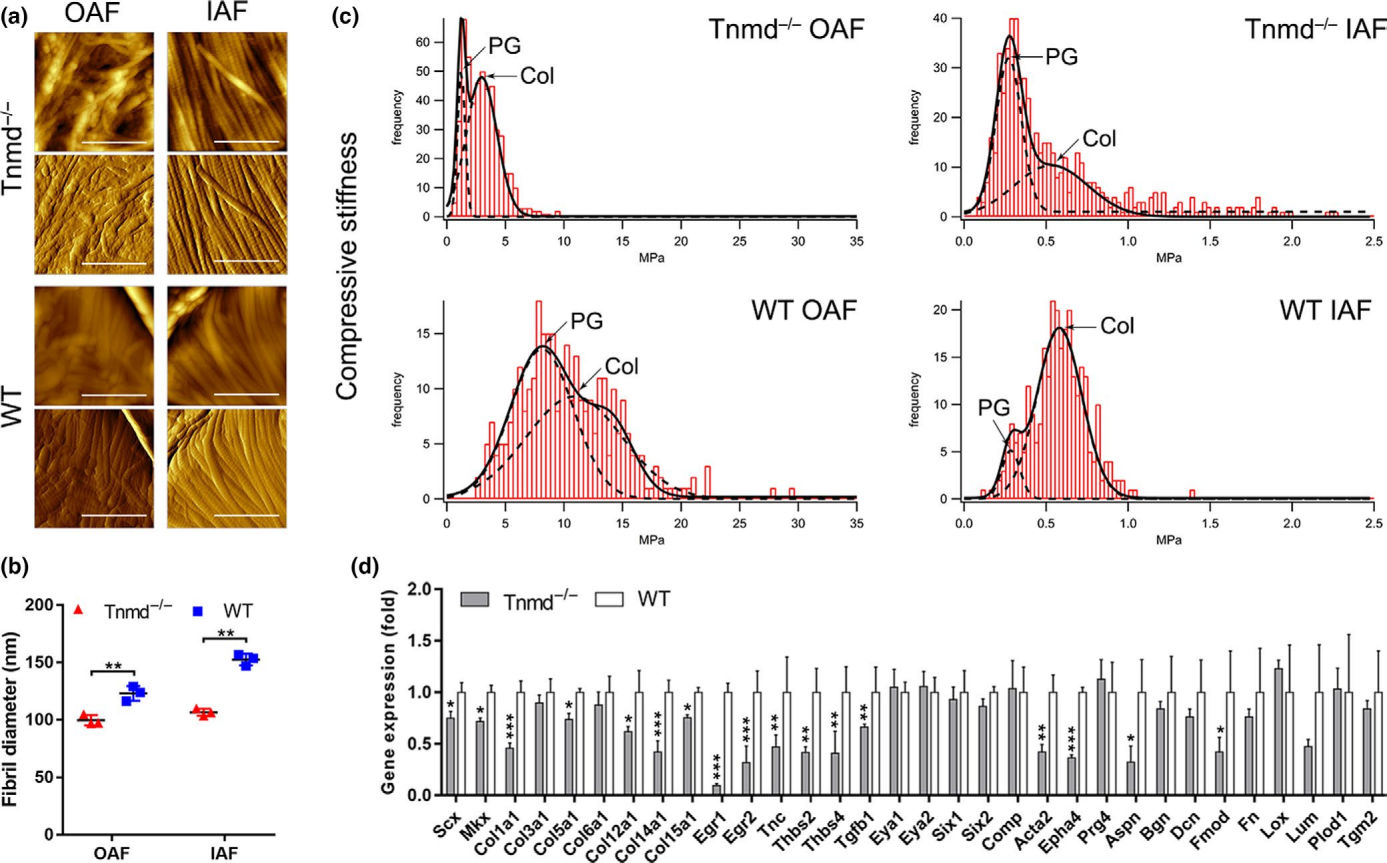
*Tnmd* deficiency causes altered ECM nanostructure and mechanical properties of the OAF in 6‐month‐old mice. (a) AFM height images (upper panels for both genotypes) show that the collagen fibrils in *Tnmd^−/−^* OAF were more frayed and interrupted by gaps, and vertical deflection images (lower panels) demonstrate that the collagen network in this region was less dense in *Tnmd^−/−^* compared to WT. (b) Comparison of the collagen fibril diameters reveals significantly smaller average size in *Tnmd^−/−^* than in WT AF (two‐tailed unpaired Student's *t* test; *n* = 3 animals, and 200 fibrils were analyzed per genotype). (c) Plots of compressive stiffness data obtained by indentation AFM demonstrated that ECM compressive stiffness was markedly lower in the OAF regions of *Tnmd^−/−^* IVDs but not noticeably different in the IAF regions (two‐tailed unpaired Student's *t* test; *n* = 3 animals). (d) Significant downregulation of numerous IVD‐ and tendon/ligament‐related genes was detected in *Tnmd^−/−^* OAF by qRT–PCR analysis. For calculation of fold changes, WT was set to 1 (two‐tailed unpaired Student's *t* test; *n* = 3 animals). ^*^ *p* < .05; ^**^ *p* < .01; ^***^ *p* < .001. AF, annulus fibrous; Col, collagen; ECM, extracellular matrix; IAF, inner annulus fibrous; IVD, intervertebral disc; OAF, outer annulus fibrous; PG, proteoglycan; qRT–PCR, quantitative real‐time PCR; WT, wild‐type. Scale bar, 1 μm

Since Tnmd is highly expressed in the OAF and a disorganized OAF morphology characterized by thinner collagen fibers (Figure S1a,b) was observed in *Tnmd* ^−^ *^/^* ^−^ mice, we next examined the nanotopographical and biomechanical properties of 6‐month‐old *Tnmd* ^−^ *^/^* ^−^ and WT IVDs. We applied indentation‐type atomic force microscopy (IT‐AFM) to quantitatively assess collagen fibril diameters and compressive stiffness of AF. Height images revealed that the collagen fibrils of *Tnmd* ^−^ *^/^* ^−^ OAF were more frayed and interrupted by gaps, and vertical deflection images indicated that the collagenous network of *Tnmd* ^−^ *^/^* ^−^ OAF was less dense compared to WT (Figure 2a). The fibril diameters in OAF and IAF were significantly smaller in *Tnmd* ^−^ *^/^* ^−^ than in WT mice (Figure 2b). Indentation measurements on native IVD tissues indicated a bimodal stiffness distribution in both genotypes (Figure 2c). In *Tnmd* ^−^ *^/^* ^−^ OAF and IAF, the proteoglycan stiffness peaks were detected at 1.240 ± 0.098 and 0.272 ± 0.004 MPa and those of collagen network at 2.972 ± 0.033 and 0.532 ± 0.049 MPa, respectively. In WT OAF and IAF, the average proteoglycan stiffness peaks were 8.134 ± 0.307 and 0.285 ± 0.013 MPa, while the collagen stiffness peaks were 14.019 ± 0.493 and 0.581 ± 0.0063 MPa, respectively. In sum, the overall compressive stiffness of OAF was markedly lower in *Tnmd* ^−^ *^/^* ^−^ than WT mice, but the IAF biomechanical properties were not significantly different between genotypes.

Changes in expression levels of ECM and cross‐linking molecules can lead to loss of mechanical properties and, thus, impaired ability of the OAF to resist compression delivered to the IVD and particularly the NP (Feng, Danfelter, Strömqvist, & Heinegård, 2006). Therefore, we analyzed how the ablation of *Tnmd* affects the expression levels of IVD‐ and tendon/ligament‐related genes using quantitative real‐time PCR (qRT–PCR) on *Tnmd* ^−^ *^/^* ^−^ and WT lumbar OAF tissue‐derived mRNA. We observed downregulation of multiple IVD‐ and tendon/ligament‐related genes including scleraxis (*Scx*), *Mkx*, collagens I, V, XII, XIV, and XV (*Col1a1*, *Col5a1*, *Col12a1*, *Col14a1*, *Col15a1*), early growth response protein 1 and 2 (*Egr1*, *Egr2*), tenascin C (*Tnc*), thrombospondin 2 and 4 (*Thbs2*, *Thbs4*), transforming growth factor beta 1 (*Tgfb1*), alpha smooth muscle actin (*Acta2*), ephrin type‐A receptor 4 (*Epha4*), asporin (*Aspn*), and fibromodulin (*Fmod*), without affecting those of collagens III and VI (*Col3a1*, *Col6a1*), eyes absent homolog 1 and 2 (*Eya1*, *Eya2*), sine oculis homeobox homolog 1 and 2 (*Six1*, *Six2*), cartilage oligomeric protein (*Comp*), lubricin (*Prg4*), biglycan (*Bgn*), decorin (*Dcn*), fibronectin (*Fn*), lysyl oxidase (*Lox*), lumican (*Lum*), procollagen‐lysine, 2‐oxoglutarate‐5‐dioxygenase 1 (*Plod1*), and transglutaminase 2 (*Tgm2*) in *Tnmd* ^−^ *^/^* ^−^ compared to WT mice (Figure 2d). Additionally, we compared the mRNA levels of IVD‐ and tendon/ligament‐related genes between tendon and OAF tissues from both genotypes, and showed that the absence of *Tnmd* in the tendon and the OAF causes opposite effects on the mRNA expression levels of *Scx*, *Mkx*, *Col14a1*, *Col15a1,* and *Prg4* (Figure S1c) suggesting tissue‐specific regulation.

Taken together, these findings demonstrate that *Tnmd* is a critical factor required to maintain the structural and biomechanical properties of the OAF collagen fibrils likely through the modulation of ECM gene expression.

### 2.4 Increased angiogenesis, macrophages infiltration, and apoptosis in Tnmd^−/−^ OAF

**Figure 3:**
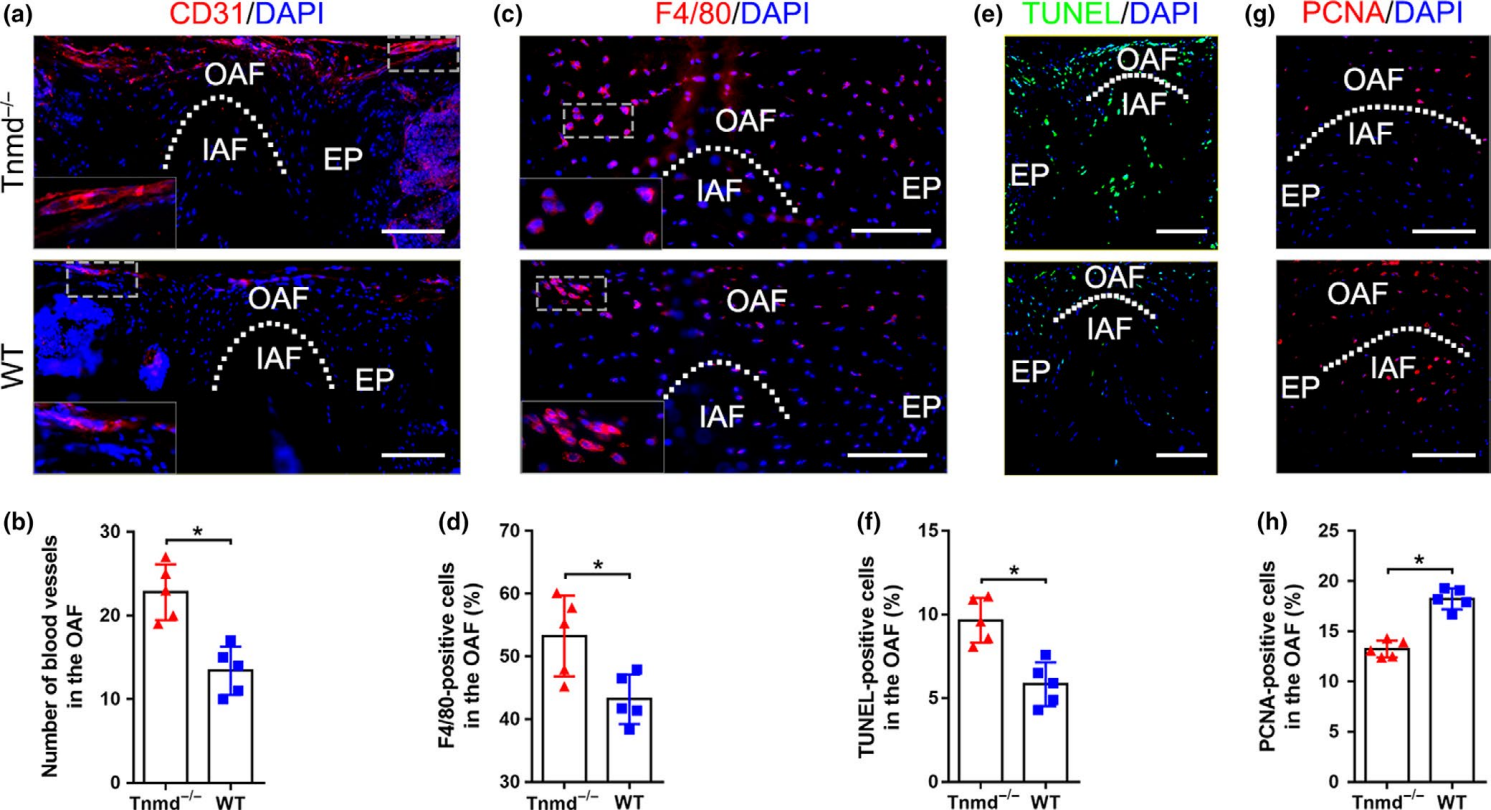
Loss of *Tnmd* results in blood vessel ingrowth, macrophage infiltration, and increased cell apoptosis in the OAF. (a and b) Immunofluorescence staining with anti‐CD31 antibody reveals increased vessel number in the OAF of *Tnmd^−/−^* than WT. (c and d) Higher number of F4/80‐positive macrophages was detected in the OAF of *Tnmd^−/−^*versus WT. (e and f) TUNEL staining demonstrates increased number of apoptotic cells in *Tnmd^−/−^* OAF than WT. (g and h) Reduced number of proliferating cells was observed in *Tnmd^−/−^* compared to WT mice by PCNA immunofluorescence staining. All quantitative histomorphometry data were assessed by two‐tailed nonparametric Mann–Whitney test; *n* = 5 animals. ^*^ *p* < .05. EP, endplate; IAF, inner annulus fibrous; OAF, outer annulus fibrous; TUNEL, transferase‐mediated dUTP‐biotin nick end labeling; WT, wild‐type; white dotted line, OAF‐IAF boundary. Scale bar, 200 μm

The AF and EP are natural barriers resistant to vascular invasion due to intrinsic angiogenic inhibitors. IVD degeneration is often marked by blood vessel ingrowth, infiltration of inflammatory cells, and increased cell apoptosis (de Vries, van Doeselaar, Meij, Tryfonidou, & Ito, 2018; Freemont et al., 1997; McCann & Séguin, 2016; Phillips, Jordan‐Mahy, Nicklin, & Le Maitre, 2013). For this reason, we then focused our investigation on the OAF zone in 6‐month‐old mice in order to reveal if *Tnmd* contributes to the maintenance of avascularity. We found that the occurrence of CD31‐labeled vessels was increased in the OAF of *Tnmd* ^−^ *^/^* ^−^ mice when compared with WT mice (Figure 3a,b). Infiltration of macrophages was confirmed by staining with F4/80 monoclonal antibody directed specifically against mouse macrophages, demonstrating a significant increase in macrophage number in the OAF zone of *Tnmd* ^−^ *^/^* ^−^ IVDs (Figure 3c,d). We also performed in situ terminal deoxynucleotidyl transferase‐mediated dUTP‐biotin nick end labeling (TUNEL) assay and immunofluorescent staining for p53, to detect if apoptotic and senescent cells are present in the OAF of *Tnmd* ^−^ *^/^* ^−^ mice, landmarks of IVD degenerative processes (Feng et al., 2016). We observed a higher number of TUNEL‐ and p53‐positive cells in *Tnmd* ^−^ *^/^* ^−^ compared to WT OAF (Figure 3e,f, Figure S1d,e). Lastly, we carried out immunofluorescent staining for the proliferative marker, PCNA, which confirmed a lower number of dividing cells in the OAF zone of 6‐month‐old *Tnmd* ^−^ *^/^* ^−^ than WT IVDs (Figure 3g,h). Taken together, the above findings indicate the essential role of the locally expressed *Tnmd* in IVD homeostasis, its loss led to induced angiogenesis, macrophage infiltration, and apoptosis, while cell proliferation was significantly reduced.

### 2.5 Manifestation of hypertrophic chondrocytes and Col X‐rich matrix in the NP of Tnmd^−/−^ mice

**Figure 4:**
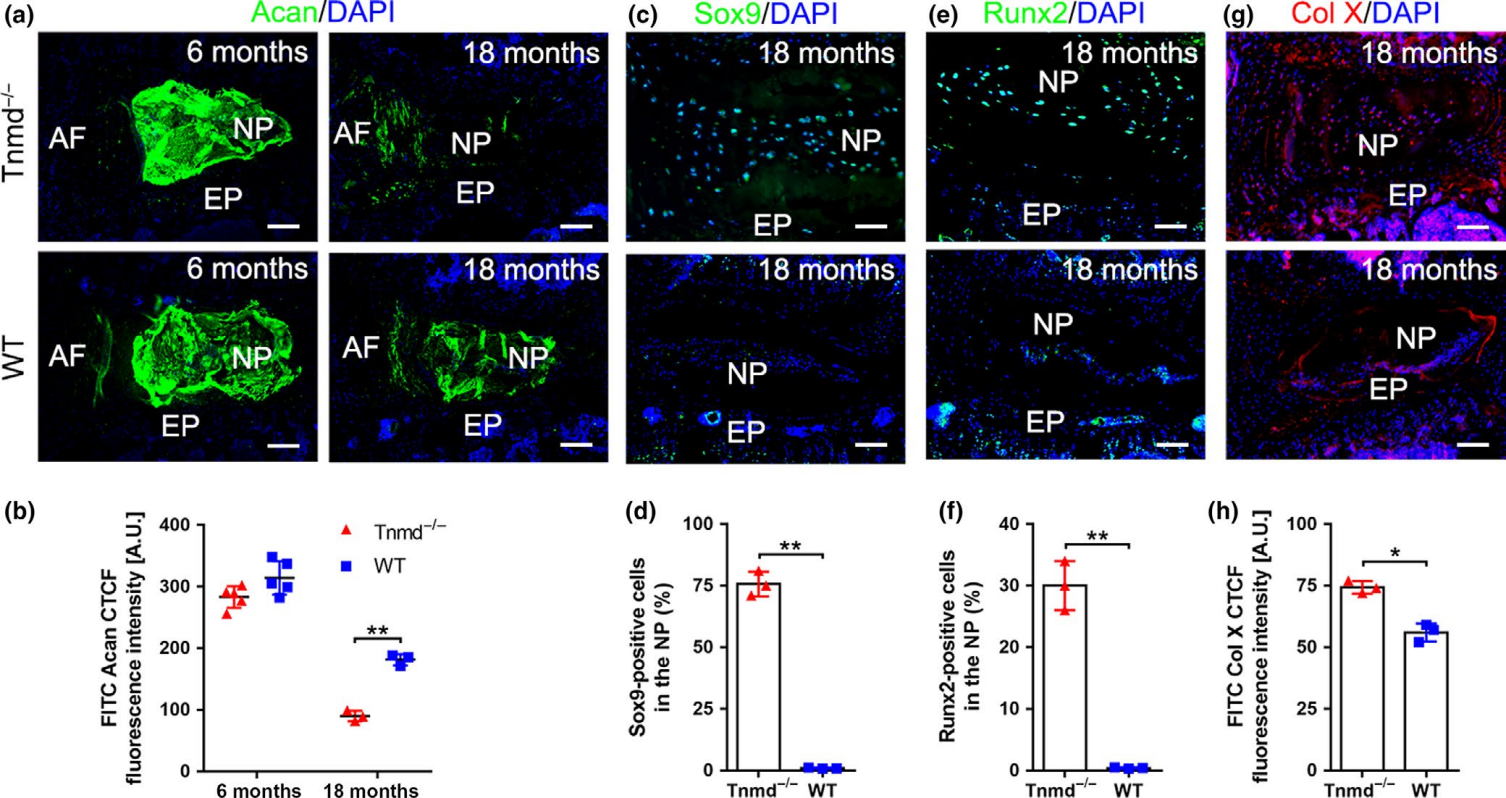
Accumulation of hypertrophic chondrocyte‐like alterations in the NP of *Tnmd^−/−^* mice. (a and b) Significant downregulation of Acan protein expression was detected at 18 months by immunofluorescent imaging and fluorescence intensity analysis in *Tnmd^−/−^* NP compared to WT. (c–h) Immunofluorescence stainings with anti‐Sox9, anti‐Runx2 and anti‐Col X antibodies reveal increased expression levels of the three hypertrophy markers in *Tnmd^−/−^* NPs compared with WT at 18 months. All quantitative histomorphometry data were assessed by two‐tailed nonparametric Mann–Whitney test; 6‐month‐old mice, *n* = 5 animals; 18‐month‐old mice, *n* = 3 animals. ^*^ *p* < .05; ^**^ *p* < .01. AF, annulus fibrous; EP, endplate; mo, month; NP, nucleus pulposus; WT, wild‐type. Scale bar, 200 μm

It has been shown that ectopic calcifications in IVDs are a known characteristic of IVD degeneration (Hristova et al., 2011; Illien‐Jünger et al., 2016). The observed small round cells, resembling chondrocyte morphology, in the *Tnmd* ^−^ *^/^* ^−^ NP prompted us to test whether the loss of *Tnmd* is accelerating hypertrophic chondrocyte‐like occurrence. The major proteoglycan of the NP is aggrecan (Acan), which due to its highly anionic glycosaminoglycan content provides osmotic properties, enabling the NP to maintain height and turgor against compressive loads (Bedore et al., 2013). Therefore, we explored Acan expression by immunohistochemistry (IHC) in IVD sections from both genotypes. At 6 months, no noticeable change in Acan localization was found in *Tnmd* ^−^ *^/^* ^−^ NP compared to WT NP. However, the deposition of Acan was significantly decreased in Tnmd‐deficient NP at 18 months (Figure 4a,b). More specifically, it has been proposed that Acan production ratio within the NP to hyaline cartilage is approximately 27:2 (Mwale, Roughley, & Antoniou, 2004). Hence, Acan downregulation can lead to appearance of hypertrophic‐like chondrocytes, which subsequently contribute to calcification, due to the available free calcium ions (Hristova et al., 2011). In order to track hypertrophic chondrocyte‐like differentiation in the NP, we next implemented immunofluorescence analysis for Sox9, the key pro‐chondrogenic transcription factor (Takimoto, Oro, Hiraki, & Shukunami, 2012), and Runx2 and Col X, markers of hypertrophic chondrocytes (van der Kraan & van den Berg, 2012). *Tnmd* ^−^ *^/^* ^−^ NP showed compositional alterations associated with increased levels of Sox9‐ and Runx2‐positive cells and Col X deposition at 18 months of age in contrast to WT mice (Figure 4c–h). These protein data together with the observed transcriptional changes of multiple genes (Figure 2d) suggested that *Tnmd* deficiency alters the balance in the expression of ECM molecules and is manifested by accumulation of hypertrophic chondrocytes and Col X‐rich matrix in the NP, indicating clearly substantial IVD cell dysfunction.

### 2.6 Tnmd and Chm1 double mutant mice display not only accelerated IVD degeneration but also ectopic bone formation

**Figure 5:**
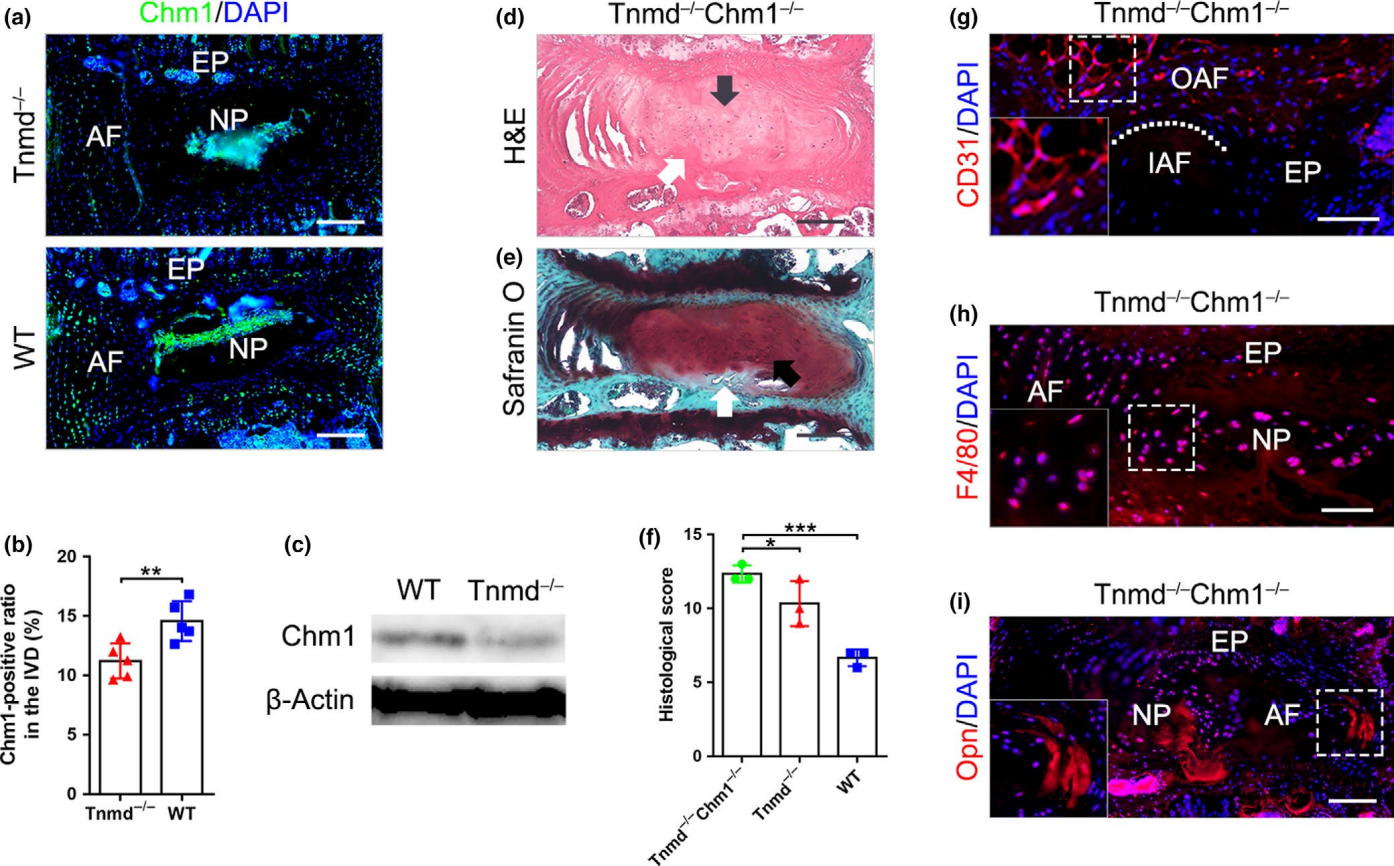
Double knockout for *Tnmd* and *Chm1* lead to accelerated IVD degeneration coupled with ectopic bone formation. (a and b) Immunofluorescence analysis of Chm1 expression shows Chm1 major localization in the NP as well as lower protein levels in *Tnmd^−/−^* than WT IVD at 6 months (two‐tailed nonparametric Mann–Whitney test; *n* = 5 animals). (c) Western blotting confirmed the downregulation of Chm1 protein in *Tnmd^−/−^* compared to WT IVD (*n* = 3 animals). (d‐f) H&E and safranin O staining reveal advanced IVD degeneration and ectopic bone formation in *Tnmd* ^−/−^ *Chm1^−/−^* mice, and histological grading shows significantly worsened scores at 18 months in *Tnmd* ^−/−^ *Chm1^−/−^* mice compared to *Tnmd* ^−/−^ and WT mice (one‐way ANOVA was followed by Bonferroni post hoc correction, *n* = 3 animals). (g) Immunofluorescence analysis for CD31 demonstrate that *Tnmd^−/−^Chm1^−/−^* OAF contain many capillary‐like structures (*n* = 3 animals). (h) Immunofluorescence analysis for F4/80 shows many macrophages were distributed in and around the NP of *Tnmd^−/−^Chm1^−/−^* mice (*n* = 3 animals). (i) Multiple sites of ectopic ossifications in *Tnmd^−/−^Chm1^−/−^* IVD were observed by carrying out Opn immunostaining (*n* = 3 animals). ^*^ *p* < .05; ^**^ *p* < .01; ^***^ *p* < .001. AF, annulus fibrous; EP, endplate; H&E, hematoxylin–eosin; IAF, inner annulus fibrous; IVD, intervertebral disc; NP, nucleus pulposus; OAF, outer annulus fibrous; WT, wild‐type; black arrows, chondrocyte‐like cells; white arrows, ectopic ossifications; white dotted line, OAF‐IAF boundary. Scale bar: 200 μm

Our findings suggest that Tnmd contributes to protect the IVD from vascularization and inflammation. However, our present data do not exclude the possibility of the existence of other anti‐angiogenic factors. Chm1, the only Tnmd homologous protein (Brandau et al., 2001; Shukunami et al., 2001), is a cartilage‐specific angiogenesis inhibitor (Hiraki et al., 1997; Yoshioka et al., 2006) that has been previously shown to be also highly expressed in the IVD during the gestational period and gradually downregulated after maturation (Takao, Iwaki, Kondo, & Hiraki, 2000). Immunofluorescence staining for Chm1 in IVD showed that it is deposited in the ECM of NP, as well as expressed in cells from EP and OAF (Figure 5a). Western blotting and densitometric analyses of Chm1‐positive areas revealed that Chm1 levels are decreased in *Tnmd* ^−^ *^/^* ^−^ IVD when compared with WTs (Figure 5b,c). These lines of evidence suggest that the expression of *Tnmd* and *Chm1* may be coordinated between the cell populations of NP, AF, and EP. Therefore, we carried out a pilot investigation of *Tnmd* ^−^ *^/^* ^−^ *Chm1* ^−^ *^/^* ^−^ mouse model to elucidate for the first time the relationship between both proteins and IVD degeneration. Interestingly, H&E and safranin O staining demonstrated that *Tnmd* ^−/−^ *Chm1* ^−^ *^/^* ^−^ mice display more severe IVD degeneration associated with ectopic bone formation in the IVDs at 18 months when compared with *Tnmd* ^−/−^ and WT mice (Figure 5d–f). Immunofluorescence of CD31 showed that the OAF of *Tnmd^−/−^Chm1^−/−^* mice contained many capillary‐like structures (Figure 5g). Furthermore, multiple F4/80‐labeled macrophages were distributed in the AF and NP regions (Figure 5h) and, lastly, ectopic ossification sites were detected with osteopontin (Opn, bone‐specific marker) antibody in *Tnmd^−/−^Chm1^−/−^* IVDs Figure 5i). Based on our novel data, we concluded that simultaneous loss of *Tnmd* and *Chm1* causes a more progressive IVD degenerative phenotype than *Tnmd* single knockout. It remains to be investigated to what extent these mutant variants compare to *Chm1* single knockout.

### 2.7 Loss of Tnmd in OAF cells suppresses their proliferation and promotes cell apoptosis

To further understand the mechanisms underlying the roles of *Tnmd* in IVD homeostasis, we performed in vitro studies with OAF cells isolated from 12‐month‐old *Tnmd^−/−^* or WT lumbar IVDs (Figure S2a). First, we characterized the obtained cell populations by immunofluorescence staining and RT–PCR. Fmod, an accepted AF‐specific marker in rodent (Leung, Tam, Chan, Chan, & Cheung, 2011; Smits & Lefebvre, 2003), was expressed in both genotypes, but its immunostaining signal was weaker in *Tnmd^−/−^* OAF cells compared with WTs (Figure S2b,g).

WT OAF cells were strongly expressing Tnmd, while as expected Tnmd was not produced by *Tnmd^−/−^* cells (Figure S2c). To analyze whether the synthesis of ECM proteins by the OAF cells is altered due to *Tnmd* deficiency, we next performed immunofluorescence analysis for Col I, which is the main protein component of OAF; Fn, which plays a pivotal role in facilitating AF cell attachment and fiber alignment (Attia, Santerre, & Kandel, 2011); and Lum, which interacts with collagen fibrils and contributes to AF mechanical properties (Sztrolovics, Alini, Mort, & Roughley, 1999). In vitro*,* only a slight decrease in the expression of the three proteins was observed in *Tnmd^−/−^* OAF cells (Figure S2d–g). Semiquantitative RT–PCR revealed that *Tnmd* mRNA could not be detected, while *Chm1* mRNA levels were reduced in *Tnmd^−/−^* OAF cells (Figure S2h).

To further evaluate the role of *Tnmd* in OAF cell behavior, we carried out time‐lapse imaging of random migration, followed by plotting of forward migration index (FMI). Our results showed that *Tnmd^−/−^* OAF cells were less migratory than WT cells (Figure S3a,b). Quantification of velocity, accumulated and Euclidean distance clearly indicated that the observed effect was significant (Figure S3c,d). Furthermore, during 0, 3, 5, and 7 days of culture, DNA‐based CyQUANT assays showed that the proliferation of *Tnmd^−/−^* OAF cells was significantly reduced compared to that of WT cells (Figure S3e). Lastly, TUNEL assays demonstrated that *Tnmd^−/−^* OAF cell population contained increased number of apoptotic cells compared to WT cell population (Figure S3f,g). Taken together, we validated the OAF phenotype of our isolated cells and concluded that the loss of *Tnmd* causes reduction in their proliferative and migratory potential but an increase in apoptotic risk.

### 2.8 Human umbilical vein endothelial cells migrate more toward Tnmd^−/−^ OAF cells which exhibit elevation in p65 and MMPs expression levels

**Figure 6:**
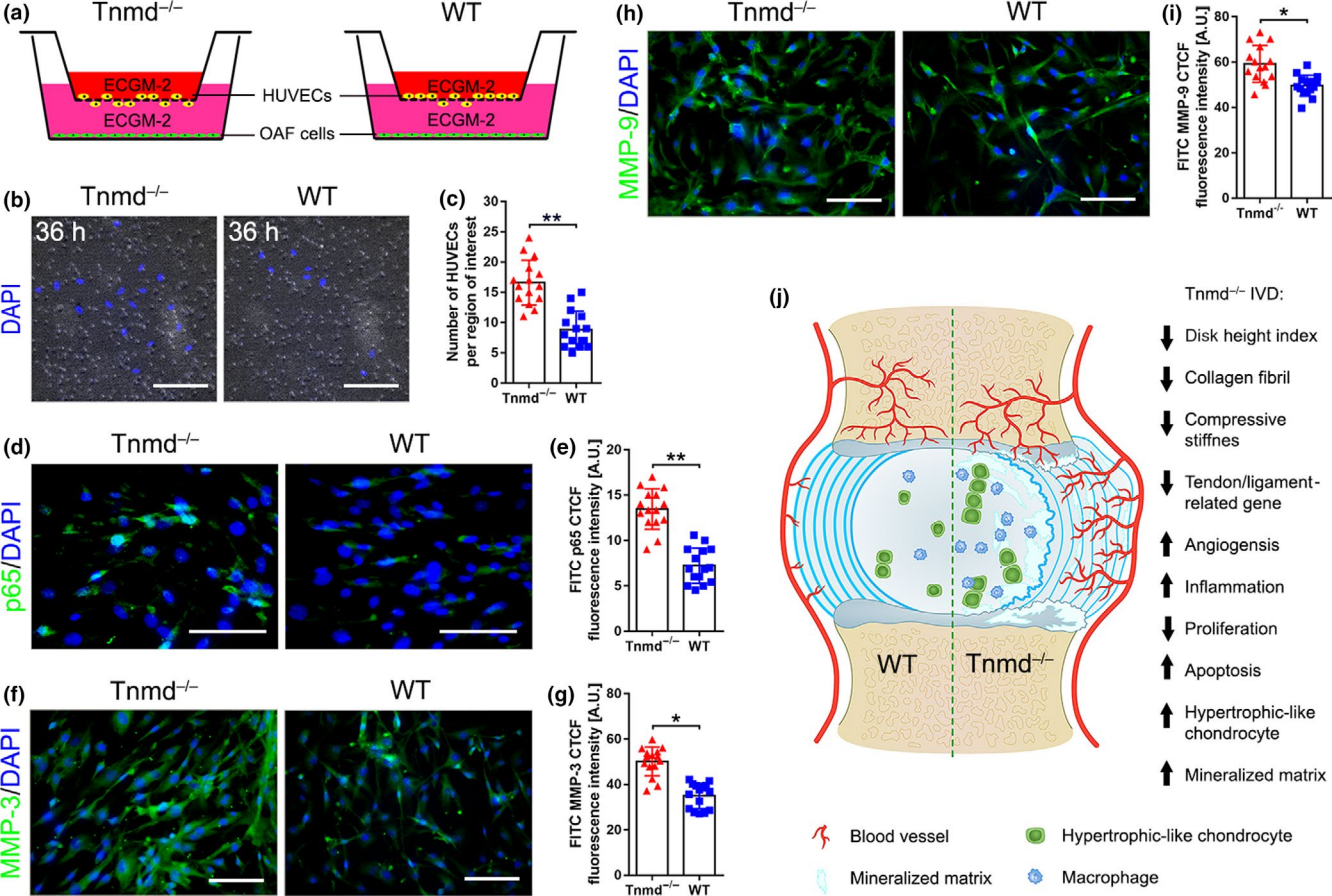
*Tnmd^−/−^* OAF cells promote HUVECs migration and have elevated p65 and MMPs expression. (a) Experimental design of HUVECs‐OAF cells co‐culture experiments. (b and c) Significantly increased migration of HUVECs toward *Tnmd^−/−^* OAF cells. Representative images of the bottom side of the transwell membrane taken after 36 hr of co‐culturing (*n* = 3 independent experiments). (d–i) *Tnmd^−/−^* OAF cells exhibit upregulated protein expression of p65 (component NF‐κB complex), MMP‐3, and MMP‐9 compared with WT cells (two‐tailed nonparametric Mann–Whitney test; *n* = 3 independent experiments). (j) Cartoon highlighting the hallmarks of *Tnmd^−/−^* IVD phenotype. ^*^ *p* < .05; ^**^ *p* < .01. Scale bar: 100 μm. HUVEC, human umbilical vein endothelial cells; IVD, intervertebral disc; MMP, matrix metalloproteinases; NF‐κB, nuclear factor‐kappa‐B; OAF, outer annulus fibrous; WT, wild‐type

Following our observation of increased angiogenesis and macrophage infiltration in *Tnmd^−/−^* OAF, we investigated the human umbilical vein endothelial cells (HUVECs) migratory capacity toward WT or *Tnmd^−/−^* OAF cells by implementing transwell assays and found that loss of Tnmd in OAF supernatant promoted their migratory capacity compared to co‐culture with WT OAF cells (Figure 6a–c). Immunofluorescence analysis revealed that the number of nuclei positive for p65, a key regulator of nuclear factor‐kappa‐B activation and function, was significantly increased in *Tnmd^−/−^* compared with WT OAF cells (Figure 6d,e), which was in parallel with elevated expression of MMP‐3 and MMP‐9 in *Tnmd^−/−^* OAF cells compared with WT OAF cells (Figure 6f–i). Thus, our results suggest that Tnmd‐deficient OAF cells exhibit elevated p65, MMP‐3, and MMP‐9 expression and that the absence of secreted Tnmd by these cells significantly promotes HUVECs migration.

## 3 DISCUSSION

Low back pain is an enormous medical and socioeconomic burden in modern society. Although there are many etiologies for the development of low back pain, a main component appears to be IVD degeneration, including neovascularization and inflammatory element (Cornejo, Cho, Giannarelli, Iatridis, & Purmessur, 2015; Freemont et al., 1997; Kwon, Moon, Kwon, Park, & Kim, 2017; Risbud & Shapiro, 2014). In the present study, we demonstrate the important role of *Tnmd* in prevention of IVD degeneration by maintaining the avascular nature of the IVD (Figure 6j).

Based on our initial investigation at the protein level, we could show that Tnmd is strongly expressed in the ECM of the OAF, while Chm1 is predominantly found in the NP. We could detect to a lower extent a signal for Tnmd also in the NP; however, this should be taken with caution considering the highly conserved C‐terminus between both proteins against which the primary antibody was raised. Moreover, we could show, similar to the Achilles tendon and CTC (Dex et al., 2016), that Tnmd processing in the IVD undergoes a proteolytic cleavage at its 16kDa C‐terminal portion. Thus, we report for the first time the expression pattern of Tnmd protein in the ECM of the IVD, but also revealing that Tnmd is downregulated at 6 months of age.

Neovascularization allows the infiltration of macrophages into the IVD triggering inflammation (Fontana, See, & Pandit, 2015; Nakazawa et al., 2018; Walker & Anderson, 2004), which in turn can further amplify the vascularization (Cornejo, Cho, Giannarelli, Iatridis, & Purmessur, 2015; Lee et al., 2011), thereby triggering a self‐perpetuating process. Here, we revealed that the absence of *Tnmd* activates angiogenesis and macrophage infiltration in the IVD in vivo, as well as HUVECs migration in an in vitro co‐culture setting with OAF‐derived cells. Furthermore, we also provide direct evidence that both *Tnmd* and *Chm1* act as anti‐angiogenic cues in the IVD due to the observed severe IVD phenotype in the double mutant animals. We detected not only pronounced vascularization and infiltration of inflammatory cells into the matrix of the IVD but also unusual calcification. These data provide a new insight into the molecular mechanisms underlying the maintenance of IVD avascularity and implicate that disruption of its homeostasis leads to pathological status.

The ECM composition and organization are very important for IVD structure and function; hence, alterations in the ECM can also contribute to IVD degeneration (Cs‐Szabo et al., 2002; Hoogendoorn et al., 2008; Liu et al., 2017; Zhang et al., 2013). In general, IVD degeneration starts with proteoglycan breakdown leading to diminished water retention and disc dehydration. Our previous nanostructural and biomechanical analyses of the collagen matrix in Achilles tendons demonstrated that the absence of *Tnmd* causes thickening and stiffening of the collagen fibrils (Docheva et al., 2005). Therefore, we analyzed whether the lack of *Tnmd* affects the collagen fibrils of the AF. In contrast to our previous results, we found out that *Tnmd^−/−^* OAF contain collagen fibrils with smaller calibers and lower compressive stiffness, which cannot provide enough strength and proper load distribution over large parts of the OAF. According to these results and our previous finding that *Tnmd* is a mechanosensitive and mechanoregulated gene (Dex et al., 2017), we propose that the above‐described phenotypic differences between tendon and IVD tissues are the consequence of the different biomechanical environments, namely IVDs are subjected mainly to compressive stress while tendons to tensile forces.

Altered OAF matrix can hinder the ability of the tissue to withstand mechanical load and in turn can increase the likelihood of tear formation (Gruber & Hanley, 1998; Guterl et al., 2013; Iatridis, Nicoll, Michalek, Walter, & Gupta, 2013; Roberts et al., 2008; Shukunami et al., 2018). Interestingly, the absence of *Tnmd* in tendon versus OAF tissues caused the opposite effect on the mRNA expression levels of several ECM genes such as *Col14a1*, *Col15a1,* and *Prg4,* as well as the transcription factors *Scx*, a direct transactivator of Tnmd (Shukunami et al., 2018), and *Mkx*, both known to regulate collagens. Furthermore, we detected elevated expression of MMP‐3 and MMP‐9 in *Tnmd^−/−^* OAF cells; thus, soluble matrix proteins can be extruded from the tissue by mechanical loading and enhance the degenerative process (Kamper et al., 2016). Notably, the glycosaminoglycan to hydroxyproline ratio within the NP of young adults is approximately 27:1, whereas the ratio within the hyaline cartilage of the same individuals is about 2:1 (Mwale et al., 2004). In our study, decreased Acan content in the *Tnmd^−/−^* NP, paralleled by increased Sox9, Runx2, and Col X levels, is indicative for a transition from a hydrated gel‐like NP to a more hypertrophic chondrocyte‐like matrix that is involved in IVD degeneration. Altogether, in such circumstances, sprouting of blood vessels in the abnormal OAF matrix and further toward the center of the IVD can facilitate ectopic ossification via intermediate hypertrophy state (Roberts et al., 2008).

Apoptosis is known to be a component of IVD degeneration (Gruber & Hanley, 1998). Previously, we have reported a reduced proliferative rate in *Tnmd^−/−^* tendon tissues and derived cells, as well as an increased number of p53‐positive cells in *Tnmd^−/−^* Achilles tendons (Alberton et al., 2015; Docheva et al., 2005). Therefore, another important point that we examined was the effect of *Tnmd* on cell proliferation and apoptosis in the IVD. Interestingly and similar to tendon tissues, our in vivo and in vitro investigations convincingly prove that *Tnmd* is a positive regulator of cell proliferation and its loss accelerates apoptosis, which in turn leads to propagation of IVD aging and degenerative process. However, our study is impeded in providing an explanation of the exact molecular mode of action of *Tnmd*, due to lack of known binding partners. Therefore, further studies are promptly required to determine the signaling pathways in which *Tnmd* participates during IVD homeostasis.

Taken together, our findings provide new insights into the protective role of *Tnmd* in IVD degeneration. Understanding the precise *Tnmd*‐dependent mechanisms can form the basis of developing new therapeutic strategies for prevention or treatment of IVD degeneration.

## 4 EXPERIMENTAL PROCEDURES

### 4.1 Animals

*Tnmd^−/−^*, *Tnmd^−/−^Chm1^−/−^*, and their WT littermates mice were used in this study. The generation of the *Tnmd^−/−^*, *Tnmd^−/−^Chm1^−/−^* mice, and their primary phenotype tendon tissues and cells were described by Docheva and co‐workers (Alberton et al., 2015; Dex et al., 2017; Docheva et al., 2005; Lin et al., 2017). All the mice were backcrossed to a C57BL/6J strain.

### 4.2 Human samples

Samples comprising 5 IVDs (Table S1) were collected from 5 patients undergoing vertebral reconstruction (IVD tissues are removed and discarded) due to burst fractures or lumbar tumors in the Orthopaedic Center of People's Liberation Army, the Affiliated Southeast Hospital of Xiamen University. There were 3 males and 2 females (21–32 years of age). All patients received magnetic resonance imaging scans to confirm intact and healthy status of the IVDs. Samples were fixed immediately after removal in 4% paraformaldehyde (PFA; Merck) overnight at 4°C and then embedded in paraffin. From each patient, an informed consent was obtained. Sample collection and experimental methods were authorized by the Ethics Committee of the Xiamen University.

### 4.3 Cell culture

Mouse OAF cells were isolated according to Nakamichi and colleagues (Nakamichi et al., 2016). Briefly, *Tnmd^−/−^* or WT mice were euthanized and lumbar discs (12‐month‐old, L1/2‐5/6, 6 animals per group) were dissected under laminar flow. The discs were trimmed, and pieces of OAF tissues were obtained, then enzymatically treated overnight with 0.04% collagenase II (Worthington) in Dulbecco's modified Eagle's medium (DMEM)/Ham's F‐12 (1:1) (Biochrom) supplemented with 10% fetal bovine serum (FBS) (Sigma‐Aldrich) and 1% penicillin/streptomycin (PS) (Biochrom). Cell suspension was filtered through 70‐µm nylon mesh (VWR International) and centrifuged at 500 g for 5 min. Isolated cells were grown in DMEM/Ham's F‐12 (1:1) with 10% FBS, 1% l‐ascorbic‐acid‐2‐phosphate (Sigma‐Aldrich), 1% minimum essential medium (MEM)‐amino acid (Biochrom), and 1% PS until day 5, at which point the medium was changed for the first time. Cells between passages 1–3 were used for experiments. HUVECs (Lonza) were cultured in the endothelial cell growth medium 2 (ECGM‐2) (PromoCell). Cells in passages 7–8 were used for experiments. Both cell types were cultured at 37°C and 5% CO_2_, kept up to 70% confluency and supplemented with fresh culture media every third day.

### 4.4 Histology, immunohistology, and histomorphometry

Mouse spines were obtained after euthanasia (newborn, 15 days, 1 month, 12 months, and 18 months of age: *n* = 3 animals; 6 months of age: *n* = 5 animals). The samples were fixed in 4% PFA overnight at 4°C. Following fixation, specimens were decalcified in 10% ethylene diamine tetraacetic acid (EDTA)/phosphate‐buffered saline (PBS) pH 8.0 (Sigma‐Aldrich, Munich, Germany) for 14 days, and embedded into paraffin or cryo‐media, and then the mouse and human samples were sectioned with 6 μm (paraffin) or 10 μm (cryo), and stained with H&E and safranin O using standard protocols. Quantification of histological score was based on a new scoring system specialized on histomorphology of mouse IVD (Tam et al., 2018). In brief, the scoring system included the following evaluation: NP structure (0 point, single‐cell mass; 1 point, cell clusters ˂50%; 2 points, cell clusters ˃50%; 3 points, matrix‐rich with little cells NP; 4 points, mineralized NP), NP clefts/fissures (0 point, none; +1 point, mild; +2 points, severe), AF/NP boundary (0 point, clear cut boundary; 1 point, round chondrocyte cells at the boundary; 2 points, loss of boundary), AF structure (0 point, concentric lamellar structure; 1 point, serpentine, widened or rounded AF lamellae; 2 points, reversal of lamellae; 3 points, undefinable lamellar structure or penetrating the NP; 4 points, mineralized or lost AF), and AF clefts/fissures (0 point, none; +1 point, mild; +2 points, severe). Quantification of disc height index was performed on H&E images (Masuda et al., 2005). In general, all histology, immunohistology, and histomorphometry experiments, unless specified otherwise in the text, were reproduced in 3 sections per sample with 5 or 3 samples per group for investigation.

For immunofluorescence staining, the sections were treated with 2 mg/ml hyaluronidase (Sigma‐Aldrich) for 30 min at 37°C in order to increase antibody permeability. After washing and blocking with 2% bovine serum albumin/PBS (Sigma‐Aldrich), primary antibodies against Acan (Abcam, 1:100, ab36861), CD31 (Abcam, 1:50, ab28364), Chm1 (Santa Cruz Biotechnology, 1:200, sc‐33563), Col I (Abcam, 1:50, ab34710), Col X (Abcam, 1:50, ab58632), Fmod (Abcam, 1:200, ab81443), Fn (Abcam, 1:50, ab2413), F4/80 (Abcam, 1:100, ab100790), Lum (Abcam, 1:50, ab168348), MMP‐3 (Novus Biologicals, 1:100, NB110‐57221), MMP‐9 (Millipore, 1:100, AB19016), Opn (Abcam, 1:200, ab8448), PCNA (Invitrogen, 1:100, 13‐3900), p53 (Abcam, 1:100, ab61241), p65 (Abcam, 1:1000, ab16502), Runx2 (Abcam, 1:200, ab102711), Sox9 (Abcam, 1:400, ab3697), and Tnmd (Metabion, PAB 201603‐00002, 1:100) were applied overnight at 4°C. Corresponding Alexa Fluor 488‐ or 546‐labeled secondary antibodies (all from Life technology) were used for 1h at room temperature. Then, sections were shortly counter‐stained with via 4′,6‐diamidino‐2‐phenylindole (DAPI) (Life technology) and mounted with fluoroshield (Sigma‐Aldrich). To analyze apoptotic cells numbers, TUNEL kit was applied following the manufacturer's instructions (Abcam, ab66110). Photomicrographs were taken on the Observer Z1 microscope equipped with the Axiocam MRm camera (Carl Zeiss). Quantitative histomorphometry was carried out via an automated quantitative image analysis according to algorithms from literature (Hsieh et al., 2016; Lin et al., 2017). In brief, using ImageJ (National Institutes of Health), the following algorithm was applied: (a) area of interest was manually designated using the “drawing/selection” tool; (b) “set measurements” for area, integrated density and mean gray value was selected from the analyze menu; and (c) lastly, the corrected total cryosections fluorescence (CTCF) representing the Acan, Col I, Col X, Fmod, Fn, Lum, MMP3, MMP‐9, p65, and Tnmd expression were exported and calculated in Excel (Microsoft) as follows CTCF = media of integrated density − (media of area of selected area × mean fluorescence). To analyze Chm1‐positive ratio of IVD, automatic color pixel quantification tool in the Adobe Photoshop CS5 software (Adobe System) was calculated in percentage to the image total pixel size. For IHC of the human IVDs, sections were deparaffinized in xylene, dehydrated in ethanol, and incubated with 0.3% hydrogen peroxide in absolute methanol for 30 min at room temperature to inhibit endogenous peroxidase. To enhance the immunoreactivity toward TNMD, the sections were treated with 0.05% citraconic anhydride (Sigma‐Aldrich) in PBS for 30 min at 60ºϹ. After washing with Tris‐HCl buffer (50 mmol/L Tris‐HCl, pH 7.6), the sections were incubated with primary antibody (Anti‐TNMD antibody, Sigma‐Aldrich, 1:100, HPA034961) at 4°C overnight, followed by corresponding biotinylated secondary antibody and horseradish peroxidase‐labeled streptavidin. The colored reaction product was developed with 3,3′‐diaminobenzidine tetrahydrochloride. Finally, the sections were counter‐stained with hematoxylin. For quantification of the TNMD‐positive area, image analysis of the OAF or NP areas stained in brown was carried out using ImageJ (TNMD‐positive area/per area of interest [%]).

### 4.5 IT‐AFM

Indentation‐type atomic force microscopy was performed on 14 μm‐thick frozen tissue sections from 6‐month‐old *Tnmd^−/−^* and WT mice (3 animals per group; each animal represented by 3 tissue sections). Measurements were taken with NanoWizard AFM instrument (JPK Instruments) mounted on an inverted optical microscope (Axiovert 200, Zeiss) as described in detail previously (Dex et al., 2017; Gronau et al., 2017; Kamper et al., 2016). Briefly, 625 indentation curves per sample were recorded in 2 × 2 μm^2^ area using pyramidal tips with nominal radius of 20 nm and silicon nitride cantilevers (MLCT microcantilever, Bruker). The spring constant of each cantilever was determined prior to the experiment using the thermal noise method, and the sample stiffness was determined via a modified Hertz model using the JPK Data Processing software (V4.2.20, JPK Instruments), as described in detail previously (Gronau et al., 2017; Kamper et al., 2016). Based on these results, histograms were plotted and the two maxima were identified by fitting a linear combination of two Gaussian functions using OriginLab software (version 6). As previously reported, the lower stiffness peak attributes to the proteoglycan moiety, while the higher stiffness peak relates to collagen fibrils (Gronau et al., 2017; Kamper et al., 2016).

### 4.6 Western blot analysis

Mouse IVDs from WT (1 and 6 months of age) and Tnmd*^−/−^* (6 months of age) animals were snap‐frozen in liquid nitrogen. Using a mortar and pestle, the tissue was pulverized and resuspended in 8 M urea, 50 mM Tris‐HCl (pH 8.0), 1 mM dithiothreitol, and 1 mM EDTA. Protein (22 μg) aliquots were loaded on a 12% SDS‐polyacrylamide gel and transferred to Immobilon‐p transfer membrane (Merck Millipore). The membrane was blocked for 90 min with 5% Milk/TBS‐T buffer. Anti‐Tnmd (1:500), anti‐Chm1 (1:500), and anti‐β‐Actin (Abcam, 1:1,000, ab8227) antibodies were added. After overnight incubation with the primary antibodies at 4°C, membranes were washed and probed with corresponding horseradish peroxidase‐conjugated goat anti‐rabbit IgG (Thermo Fischer Scientific). Protein bands were visualized using SuperSignal West Dura Extended Duration Substrate (Thermo Fischer Scientific) and film paper. Western blot was independently reproduced with two separate preparation of IVDs from 1‐ and 6‐month‐old *Tnmd^−/−^* and WT animals.

### 4.7 Semiquantitative and qRT–PCR

Total RNAs from OAF tissues (*n* = 3 animals) and OAF cells were isolated with Qiagen RNeasy Mini kit (Qiagen) and used for standard semiquantitative and qRT–PCR. For cDNA synthesis, 1 μg total RNA and AMV First‐Strand cDNA Synthesis Kit (Invitrogen) were implemented. Semiquantitative PCR was performed with Taq DNA Polymerase (Invitrogen) in MGResearch instrument (BioRad, Munich, Germany). Primer sequences and PCR conditions were as follows: *Tnmd* forward 5′gaaaccatggcaaagaatcctccagag3′, reverse 5′ttagactctcccaagcatgcgggc3′; *Chm1* forward 5′atggtagggcctgaggacgttg3′, reverse 5′gctgcatggcatgacgactctg3′; *Gapdh* forward 5′gagaggccctatcccaactc3′, reverse 5′gtgggtgcagcgaactttat3′; PCR was performed with incubation at 94°C for 5 min followed by 30 cycles of a three‐step temperature program of 1 min at 94°C, 20 s at 57°C, and 30 s at 72°C. The PCR reaction was terminated after a 7 min extension at 70°C. The band intensity of the amplified products in the gel was visualized, photographed, and analyzed using a gel imager (Vilber Lourmat). The relative gene expression was quantified by densitometry and normalized to the amount of *Gapdh* with ImageJ and presented as fold change to WT. Quantitative PCR of tendon‐associated genes was performed using RealTime Ready Custom Panel 96 – 32+ plates (https://configurator.realtimeready.roche.com) according to the manufacturer's instructions (Roche Applied Science). Briefly, PCR reactions were pipetted on ice and each well contained 10 µl LightCycler 480 probes master mix, 0.2 µl cDNA (diluted 1:5) and 9.8 µl PCR grade water. Plates were subsequently sealed and centrifuged down for 15 s. at 2,100 rpm. The relative gene expression was calculated as a ratio to *Gapdh*. All PCR results have been reproduced in three independent experiments.

### 4.8 Migration analysis

Migration analysis was performed similarly to our previous study (Popov, Kohler, & Docheva, 2016). For random migration, 1.5 × 10^3^ cells/cm^2^ of *Tnmd^−/−^* and WT OAF cells were seeded on Col I‐coated (20 μg/Ml; Millipore) 6‐well plates and incubated for 2 hr prior imaging. Time lapse was performed with 4 frames per 20 min for 24 hr. The image data were extracted with AxioVisionLE software (Carl Zeiss), and individual cell tracks were analyzed with ImageJ. Random migration was expressed by calculation of the forward migration index (FMI; the ratio of the vector length to the migratory starting point), velocity, and accumulated (cumulative track length) and Euclidian (the ordinary straight‐line length between two points) distances. Results of random OAF cells migration measurements consist of 3 independent time‐lapse movies of two *Tnmd^−/−^* and WT OAF cells donors as a total number of 20–25 OAF cells per genotype were tracked.

### 4.9 CyQUANT assays

A total of 1.5 × 10^3^ cells (passage 1) per well were plated in 6‐well plates, and CyQUANT assay detection was performed according to the manufacturer's instructions (Invitrogen) after 0, 3, 5, and 7 days cell culture. CyQUANT assay was repeated independently in 3 experiments per time point.

### 4.10 HUVECs‐OAF cells co‐culture

Co‐cultures were performed using Boyden chambers with membrane containing 8.0 μm pores inserted in 24‐well plates (Becton Dickinson Labware) as described previously (Kimura et al., 2008). In brief, *Tnmd^−/−^* or WT OAF cells (1 × 10^4^ cells per well) were seeded on the bottom of the wells and cultured in DMEM/Ham's F‐12 (1:1) with 10% FBS, 1% l‐ascorbic‐acid‐2‐phosphate, 1% MEM‐amino acid, and 1% PS for 24 hr, and then, the medium was replaced with ECGM‐2. Before seeding HUVECs into the upper chamber, the membrane was coated with Col I (10 μg/Ml; Millipore), kept in a humidified incubator overnight, and filled with ECGM‐2 an hour before introducing the 5 × 10^3^ HUVECs per well. After 36 hr co‐culturing, HUVECs that have migrated through the pores and adhered to the lower side of the membrane were fixed with 4% PFA and stained with DAPI. The cell nuclei were counted using the Observer Z1 microscope equipped with the Axiocam MRm camera. *Tnmd^−/−^* or WT OAF cells in the lower chamber were analyzed for p65, MMP‐3, and MMP‐9 by immunofluorescence staining. The interactions of the co‐cultures assays were repeated independently in 3 experiments. All cell nuclei (DAPI) in 3 images per well from 5 wells per plate were counted.

### 4.11 Statistical analysis

In this study, each animal was represented with 3 different tissue sections with comparable planes between genotypes. The results were averaged per animal were presented as mean ± *SD* between the 3–5 animals per group. Exact animal number and experimental reproducibility is given for each result in the figure legends. Statistical differences between two groups were determined using two‐tailed unpaired Student's *t* test, or two‐tailed nonparametric Mann–Whitney test. In multiple comparisons, one‐way ANOVA was followed by Bonferroni post hoc correction. Differences were considered statistically significant at the values of ^*^ *p* < .05, ^**^ *p* < .01, and ^***^ *p* < .001.

## CONFLICT OF INTEREST

The authors declare no conflict of interest.

## AUTHOR CONTRIBUTIONS

D.L. designed, performed, and analyzed experiments and wrote the manuscript; P.A. performed co‐culture experiments; C.P. and H.C‐S. carried out AFM analyses; M.D.C. performed Western blotting and RT–PCR; J.D., A.A., C.S. and J.C.I. approved manuscript; D.D. conceived the study, designed, and analyzed experiments and wrote the manuscript.

## ETHICS APPROVAL

Mouse husbandry, handling, and euthanasia were strictly carried out according to the guidelines of the Bavarian authorities. Animals were euthanized with CO_2_ and dissected for collection of whole spine and tail tendon tissues. Human sample collection and experimental methods were authorized by the Ethics Committee of the Xiamen University.
