# Supplementary material for: Loss of tenomodulin expression is a risk factor for age‐related intervertebral disc degeneration
Source: Aging Cell. 2020 Feb 21;19(3):e13091. doi: 10.1111/acel.13091 (PMC7059137; doi:10.1111/acel.13091)
Supplement: Supplementary file 1 [file ACEL-19-e13091-s001.docx]

**SUPPORTING INFORMATION**

**FIGURE S1** The absence of *Tnmd* leads to morphological changes, differential expression of IVD- and tendon/ligament-related genes and p53 in the OAF. (a and b) HE staining indicates a narrowed diameter of collagen fibrils in *Tnmd^-/-^* OAF and IAF compared to WT at 6 months of age (two-tailed non-parametric Mann-Whitney test; n=5 animals). (c) The absence of *Tnmd* in the OAF *versus* tendon tissues causes the opposite effect on the mRNA expression levels of *Scx*, *Mkx*, *Col14a1*, *Col15a1* and *Prg4* (n=3 independent experiments). (d and e) Increased number of p53-positive cells was found in the OAF of *Tnmd^-/-^* compared to WT at 6 months based on immunofluorescence analysis (two-tailed non-parametric Mann-Whitney test; n=5 animals). ^**^p<0.01. IAF, inner annulus fibrous; mo, month; OAF, outer annulus fibrous; black solid line, width of the OAF and IAF collagen fiber; black and white dotted line, OAF-IAF boundary. Scale bar, 100 μm.

**FIGURE S1**


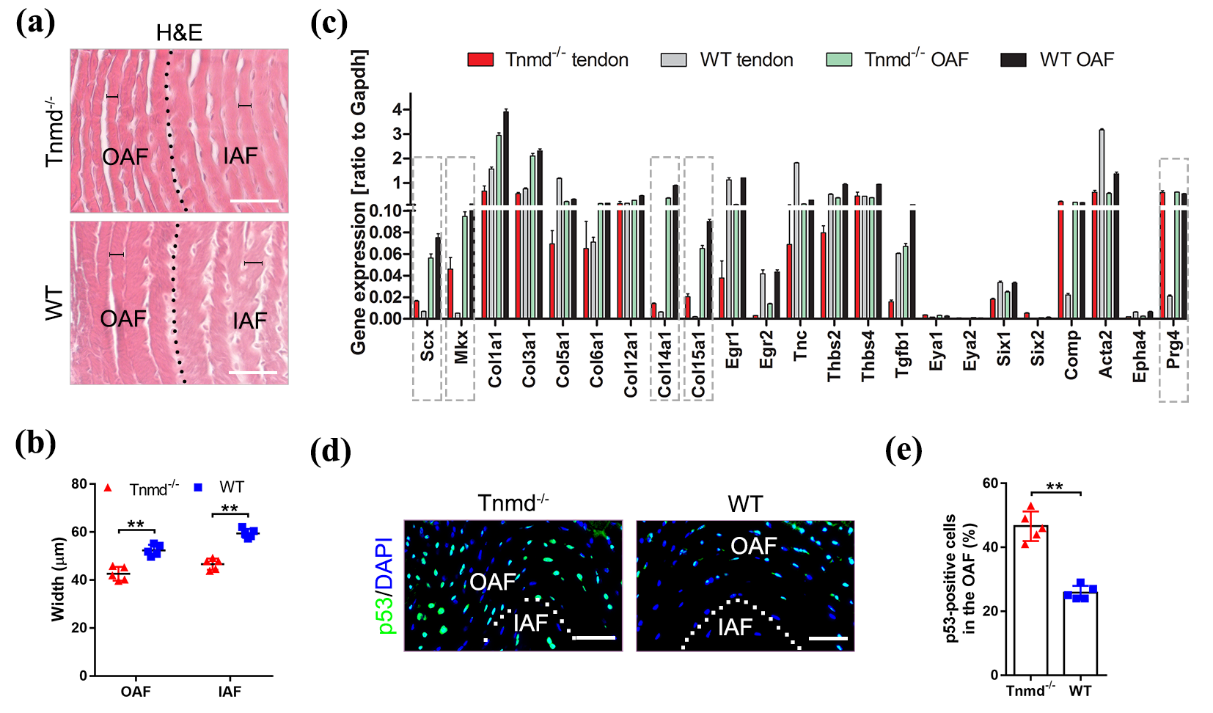


**FIGURE S2** Characterization of OAF-derived cells. (a) Phase-contrast images of isolated OAF cells from both genotypes. (b-f) Immunofluorescence staining for OAF-related matrix proteins Fmod, Tnmd, Col I, Fn, and Lum in *Tnmd^-/-^* and WT OAF cells. (g) Immunofluorescence intensity analysis (two-tailed non-parametric Mann-Whitney test; n=5 independent experiments). (h) Semiquantitative RT-PCR for *Tnmd* and *Chm1* mRNA expression in OAF cells from *Tnmd^-/-^* and WT cells (n=3 independent experiments). ^*^p<0.05. Scale bar, 100 μm.

**FIGURE S2**


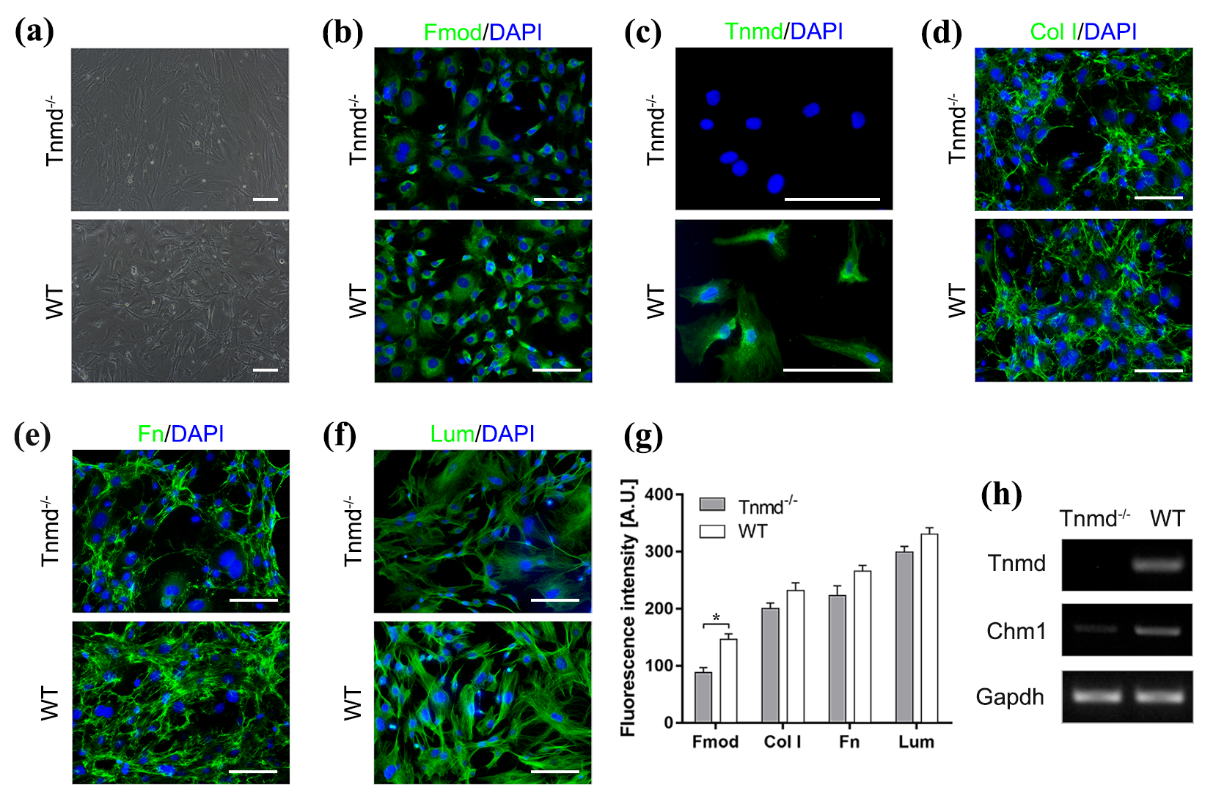


**FIGURE S3** *In vitro* investigation of OAF cells migration, proliferation and apoptosis. (a and b) Representative images of random migration and FMI plots show that *Tnmd^-/-^* OAF cells are less migratory than WT cells. Asterisks in left images show the migratory start point, while asterisks in the right images indicate the end point. (c and d) Quantification of velocity, accumulated and Euclidean distance clearly demonstrate the significant lesser migratory capacity of *Tnmd^-/-^* OAF cells (two-tailed unpaired Student’s *t* test; n=3 independent experiments). (e) Cell growth kinetics were estimated by DNA-based CyQUANT assay at day 0, 3, 5 and 7 of culture and revealed significantly lower proliferation of *Tnmd^-/-^* OAF cells compared to WT (two-tailed unpaired Student’s *t* test; n=3 independent experiments). (f and g) Increased number of TUNEL-positive cells, indicative of apoptosis, in *Tnmd^-/-^* OAF cells cultures (two-tailed non-parametric Mann-Whitney test; n=5 independent experiments). ^*^p<0.05; ^***^p<0.001. FMI, forward migration index; h, hour. Scale bar, 100 μm.

**FIGURE S3**


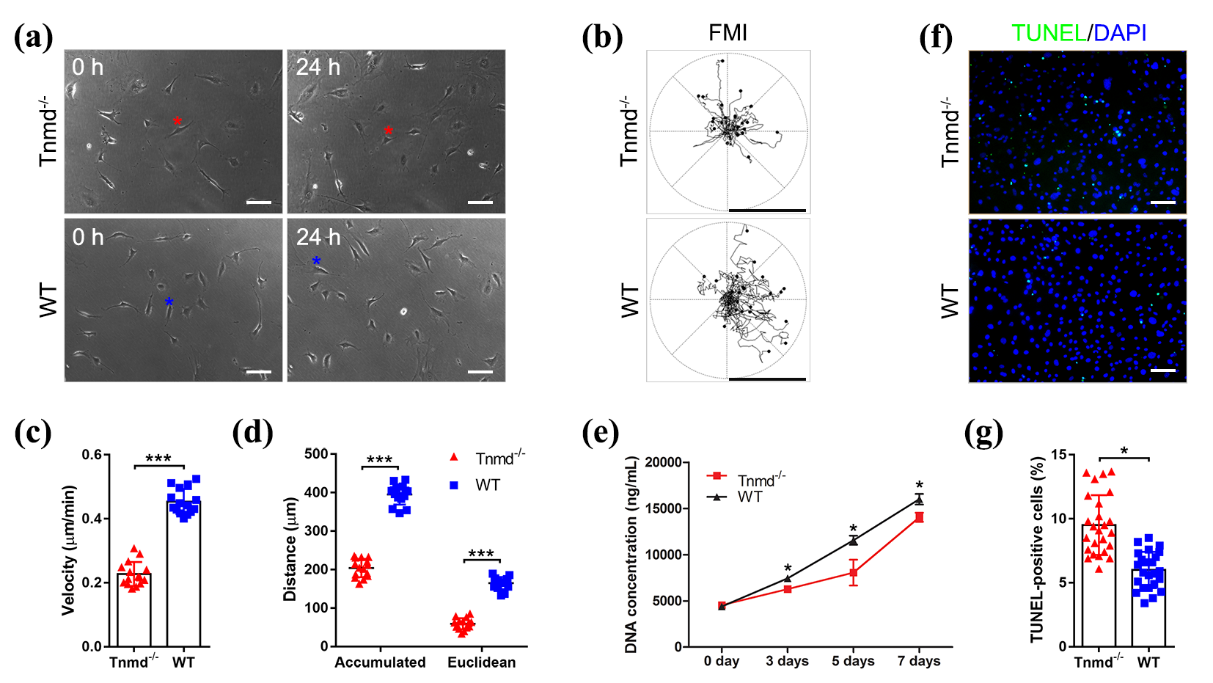


**Full uncut gels**

**Full unedited gel for Figure 1c and Figure 5c**

Legend: Total protein extracts from IVD tissues obtained in two independent preparation (1 and 6 month WT and Tnmd^-/-^ animals, lane 1-3 pool of 3 animals per genotype; lane 4-5 single animal preparation) were loaded in lanes 1-5. Total protein from tendon tissues from WT and Tnmd^-/-^ animals was loaded in lane 6 and 7, respectively. Membrane was probed with rabbit polyclonal antibody recognizing the C-terminus of Tnmd as well as that of the homolog Chm1 (Metabion, PAB 201603-00002). Upper band (approx. 22 kD) corresponds to cleaved Chm1 C-terminus; lower band (approx. 16 kD) corresponds to cleaved Tnmd C-terminus. In lane 6 only cleaved Tnmd C-terminus is detected since in tendon tissues and Chm1 is not expressed. Full length Tnmd protein (40-45 kD) was not detected suggesting complete processing of Tnmd in IVDs.

**
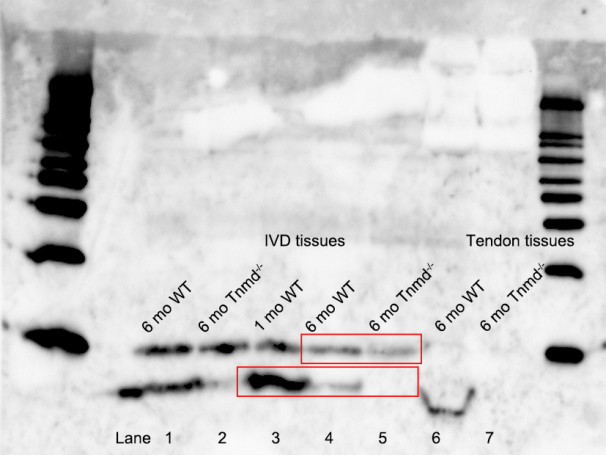
**

**Full unedited gel for Figure 1c and Figure 5c**

**Legend:** Probing of the membrane with anti-β-actin antibody for validation of protein loading. Band size approx. 45 kDa.

**
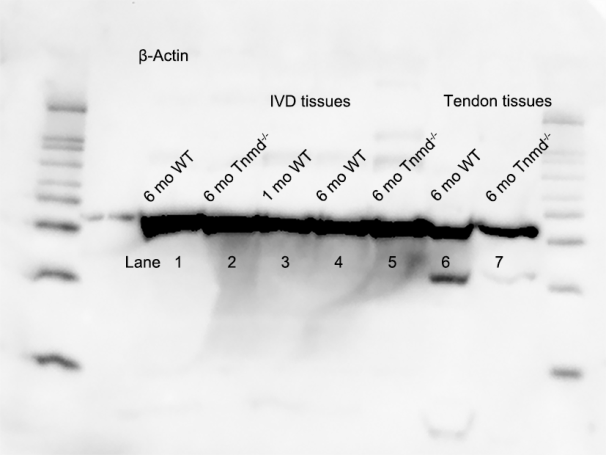
**

**TABLE S1** List of human lumbar discs.

| *#* | Sex | Age | Diagnosis | Surgery | Level | [Pfirrmann grade](https://www.ncbi.nlm.nih.gov/pubmed/30710732) |
| --- | --- | --- | --- | --- | --- | --- |
| 1  2  3  4  5 | Male  Female  Male  Female  Male | 21  27  23  28  32 | L1 burst fracture  L2 burst fracture  L3 giant cell tumor  L4 giant cell tumor  L4 plasmacytoma | Vertebral reconstruction  Vertebral reconstruction  Vertebral reconstruction  Vertebral reconstruction  Vertebral reconstruction | L1/2  L2/3  L3/4  L3/4  L4/5 | I  II  I  I  II |

**FIGURE 1b**

|  | **NB** | **15 d** | **1 mo** | **6 mo** | **12 mo** | **18 mo** |
| --- | --- | --- | --- | --- | --- | --- |
| **OAF** | 165.43 | 320.12 | 338.72 | 239.78 | 148.73 | 102.63 |
|  | 171.72 | 359.94 | 361.54 | 244.59 | 168.22 | 108.55 |
|  | 188.05 | 385.81 | 385.83 | 255.48 | 177.49 | 123.74 |
|  |  |  |  | 267.98 |  |  |
|  |  |  |  | 289.05 |  |  |
| **NP** | 94.81 | 145.75 | 234.8 | 189.04 | 85.31 | 55.94 |
|  | 97.78 | 166.94 | 262.32 | 208.56 | 93.64 | 59.98 |
|  | 105.22 | 181.21 | 295.04 | 210.58 | 99.62 | 70.21 |
|  |  |  |  | 230.14 |  |  |
|  |  |  |  | 235.98 |  |  |

FITC Tnmd CTCF: data for each animal per time point.

**FIGURE 2d**

|  | **Scx** | **Mkx** | **Col1a1** | **Col3a1** | **Col5a1** | **Col6a1** | **Col12a1** | **Col14a1** |
| --- | --- | --- | --- | --- | --- | --- | --- | --- |
| **Tnmd-/-** | 0.0498 | 0.1024 | 3.1042 | 1.9042 | 0.2146 | 0.1297 | 0.2956 | 0.3577 |
|  | 0.0627 | 0.0945 | 2.7412 | 2.2431 | 0.2498 | 0.1608 | 0.2741 | 0.4015 |
|  | 0.0564 | 0.0873 | 2.9781 | 2.1747 | 0.239 | 0.1539 | 0.2804 | 0.3752 |
| **WT** | 0.0812 | 0.1411 | 4.1127 | 2.4462 | 0.3501 | 0.1745 | 0.496 | 0.921 |
|  | 0.0685 | 0.1245 | 3.6982 | 2.3017 | 0.2983 | 0.159 | 0.4428 | 0.8874 |
|  | 0.0752 | 0.1304 | 3.9009 | 2.1876 | 0.337 | 0.1604 | 0.4706 | 0.8712 |
|  | **Col15a1** | **Egr1** | **Egr2** | **Tnc** | **Thbs2** | **Thbs4** | **Tgfb1** | **Eya1** |
| **Tnmd-/-** | 0.0605 | 0.1204 | 0.0149 | 0.1465 | 0.4012 | 0.388 | 0.0721 | 0.0033 |
|  | 0.0704 | 0.1169 | 0.0129 | 0.1288 | 0.3894 | 0.4102 | 0.0624 | 0.0026 |
|  | 0.0642 | 0.1068 | 0.0137 | 0.1359 | 0.36 | 0.3641 | 0.0662 | 0.0034 |
| **WT** | 0.0901 | 1.2045 | 0.0473 | 0.301 | 0.9871 | 0.9354 | 0.1124 | 0.0028 |
|  | 0.0864 | 1.199 | 0.0422 | 0.2874 | 0.882 | 0.9622 | 0.0987 | 0.0021 |
|  | 0.0934 | 1.1963 | 0.0407 | 0.2941 | 0.9325 | 0.9214 | 0.0939 | 0.0026 |
|  | **Eya2** | **Six1** | **Six2** | **Comp** | **Acta2** | **Epha4** | **Prg4** | **Aspn** |
| **Tnmd-/-** | 0.0013 | 0.0265 | 0.0008 | 0.2138 | 0.6047 | 0.0026 | 0.6472 | 0.0672 |
|  | 0.0009 | 0.0234 | 0.0011 | 0.1987 | 0.5123 | 0.0025 | 0.6111 | 0.0892 |
|  | 0.0011 | 0.0246 | 0.0009 | 0.2004 | 0.5834 | 0.0021 | 0.5973 | 0.1575 |
| **WT** | 0.0009 | 0.0331 | 0.0018 | 0.1901 | 1.5043 | 0.0054 | 0.5786 | 0.2101 |
|  | 0.0008 | 0.0314 | 0.0014 | 0.1885 | 1.3574 | 0.0075 | 0.5537 | 0.1983 |
|  | 0.0009 | 0.0347 | 0.0011 | 0.2014 | 1.2498 | 0.0062 | 0.5104 | 0.2384 |
|  | **Bgn** | **Dcn** | **Fmod** | **Fn** | **Lox** | **Lum** | **Plod1** | **Tgm2** |
| **Tnmd-/-** | 0.0288 | 0.3212 | 1.6343 | 1.3342 | 0.0932 | 0.7463 | 0.0653 | 0.0323 |
|  | 0.0262 | 0.3923 | 1.2134 | 0.8428 | 0.1194 | 0.8104 | 0.0887 | 0.0294 |
|  | 0.0219 | 0.3629 | 1.1249 | 0.9651 | 0.1238 | 0.7792 | 0.0759 | 0.0305 |
| **WT** | 0.0384 | 0.4239 | 1.7343 | 1.4326 | 0.0742 | 1.0609 | 0.0732 | 0.0405 |
|  | 0.0291 | 0.5885 | 1.9348 | 1.5381 | 0.0814 | 1.0754 | 0.0834 | 0.0278 |
|  | 0.0274 | 0.3629 | 2.0483 | 1.3025 | 0.8476 | 1.7454 | 0.0583 | 0.0342 |

QRT-PCR data for each animal: delta CT values.
